# Gaps in the Identification of Child Race and Ethnicity in a Pediatric Emergency Department

**DOI:** 10.5811/westjem.59145

**Published:** 2023-05-03

**Authors:** Colleen K. Gutman, K. Casey Lion, Lauren Waidner, LaShaun Bryan, Anna Sizemore, Carolyn Holland, Cindy Montero, Rosemarie Fernandez

**Affiliations:** * University of Florida, Department of Emergency Medicine, Gainesville, Florida; † University of Florida, Department of Pediatrics, Gainesville, Florida; ‡ University of Washington, Department of Pediatrics, Seattle, Washington; § Center for Child Health, Behavior, and Development, Seattle Children’s Research Institute, Seattle, Washington; || University of Florida College of Medicine, Center for Experiential Learning and Simulation, Gainesville, Florida

## Abstract

**Introduction:**

Race and ethnicity are social constructs that are associated with meaningful health inequities. To address health disparities, it is essential to have valid, reliable race and ethnicity data. We compared child race and ethnicity as identified by the parent with that reported in the electronic health record (EHR).

**Methods:**

A convenience sample of parents of pediatric emergency department (PED) patients completed a tablet-based questionnaire (February–May 2021). Parents identified their child’s race and ethnicity from options within a single category. We used chi-square to compare concordance between child race and ethnicity reported by the parent with that recorded in the EHR.

**Results:**

Of 219 approached parents, 206 (94%) completed questionnaires. Race and/or ethnicity were misidentified in the EHR for 56 children (27%). Misidentifications were most common among children whose parents identified them as multiracial (100% vs 15% of children identified as a single race, P < 0.001) or Hispanic (84% vs 17% of non-Hispanic children, P < 0.001), and children whose race and/or ethnicity differed from that of their parent (79% vs 18% of children with the same race and ethnicity as their parent, P < 0.001).

**Conclusion:**

In this PED, misidentification of race and ethnicity was common. This study provides the basis for a multifaceted quality improvement effort at our institution. The quality of child race and ethnicity data in the emergency setting warrants further consideration across health equity efforts.

## INTRODUCTION

Race and ethnicity are social constructs that are associated with meaningful health inequities. To identify and address health disparities, it is necessary to have accurate race and ethnicity data. In 2009, the Institute of Medicine released *Race, Ethnicity, and Language Data: Standardization for Healthcare Quality Improvement*, with recommendations and best practices for race and ethnicity data collection.^1^ However, inaccuracies in race and ethnicity data persist in hospital and administrative databases of adult patients.^2^–^4^

The causes of inaccuracies in race and ethnicity data are multifactorial. Although self-identified race and ethnicity are considered the gold standard by the Institute of Medicine, patients are not always given the ability to provide self-identified demographics.^1^,^5^–^7^ This may be more likely in the emergency department (ED), where hospital registration staff must find time to collect patient information and sign consents to care without interrupting urgent and fragmented clinical care. Race and ethnicity that are determined based on staff observation may be particularly inaccurate for children, who may not have the same race and/or ethnicity as their caregiver.^8^,^9^

Inaccuracies can be further compounded by limitations in available race and ethnicity categories.^1^,^9^,^10^ Healthcare systems often restrict race and ethnicity data collection to the minimum standard categories required for federal reporting and rarely give the option to select “other” or to select multiple races or “multiracial.”^11^ Additionally, although the Institute of Medicine supports the option of presenting race and ethnicity within a single question, healthcare systems often separate these into distinct categories as used in federal reporting.^1^ This format can lead to misidentification of Hispanic individuals who do not otherwise identify with the options listed in a distinct race category.^1^,^12^ As part of a quality initiative to improve and standardize accurate demographic data collection, we sought to assess the accuracy of child race and ethnicity data in an academic pediatric emergency department (PED), and to identify risk factors for misidentification.

## METHODS

We conducted a cross-sectional analysis comparing child race and ethnicity reported by the parent to that documented in the electronic health record (EHR). This study took place in a single, academic PED with an affiliated onsite children’s hospital. The PED has an annual volume of approximately 26,000 patients. In the EHR (Epic Systems Corporation; Verona, WI), race and ethnicity data are documented separately using the minimum US Office of Management and Budget categories.^7^ Both fields are required. In each field multiple options can be selected, including an option for “other.” If not already documented from a prior visit within the hospital system, PED registration staff obtain patient race and ethnicity from patient or parent self-report or through staff observation.

Between February–May 2021, three trained research assistants (RA) approached a convenience sample of parents or caregivers (“parents”). Parents of critically ill children were excluded. The RAs were in the PED during afternoons and evenings. Sample size was determined by RA availability during the study period. Parents were approached at any time during the PED visit. The RAs explained the purpose of the study and asked parents to complete a brief, tablet-based questionnaire while the RA remained in the room. Parents who used a language other than English completed the questionnaire verbally with video interpretation. The parent was asked to identify the child’s race and ethnicity from a single question (“What is your child’s race and ethnicity? You can choose as many answers as you want to: American Indian/Alaska Native; Asian; Black/African American; Hispanic/Latino; Native Hawaiian/other Pacific Islander; White; other (free-text optional); I don’t want to say; I don’t know”).^1^ We chose to use a single question with race and ethnicity presented together, an option suggested by the Institute of Medicine, so that parents were not compelled to make selections within a category with which they or their child did not identify.^1^,^8^ Parents were also asked to identify their own race and ethnicity in a similar, single-question format.^1^ Child race and ethnicity were abstracted from the EHR. This study was approved by the University of Florida Quality Improvement Project Registry and determined not to require institutional review board review.

Our primary outcome was EHR misidentification of child race and ethnicity. Child race and ethnicity was considered “misidentified” if the EHR record did not match the parent report. Misidentifications in the EHR included situations in which the parent selected multiple options but not all of those were selected in the EHR, or vice versa. This also included situations in which the parent selected “other” but “other” was not reported in the EHR, or vice versa. Finally, race and ethnicity data are often missing from EHRs, leading to the exclusion of those individuals from equity-focused analyses and research.^3^ Thus, if EHR race and/or ethnicity was missing but the parent provided a response in the questionnaire, this was considered a misidentification. To assess this approach, we conducted sensitivity analyses in which we excluded those patients with missing race and/or ethnicity. We performed statistical analyses in R (R Core Team, 2021, R Foundation for Statistical Computing, Vienna, Austria). We performed chi-square and bivariate regression analyses to evaluate misidentification by child race and/or ethnicity and age, respectively. We assessed statistical significance at the *P* = 0.05 level.

## RESULTS

Of 219 approached parents, 206 completed a questionnaire (94%). Most parents identified their child as non-Hispanic White (51%) or non-Hispanic Black (26%) (Table 1). Thirty-one parents (15%) identified their child as Hispanic, half of whom did not identify a separate race for their child. Seventeen parents (8.3%) identified their child as multiracial.

Fifty-six children (27%) had misidentified race and/or ethnicity in the EHR. Most misidentifications (89%) were misidentification of race. This includes all 17 multiracial children, most of whom were inaccurately reported as having a single race. Of the 16 Hispanic children who did not have separate race identified by the parent, 70% were misidentified as “other race,” and 30% were misidentified as “White” in the EHR. Six children had misidentified ethnicity, all of whom were identified as Hispanic by the parent and in the EHR as “not Hispanic or Latino.” A full list of misidentified EHR race and ethnicity data is found in Table 2.

Misidentifications were most common among children who were multiracial (100%, 95% confidence interval [CI] 77–100%] vs 15%, 95% CI 10–21%) of children identified as a single race, *P* < 0.001), children who were Hispanic (87%, 95% CI 69–96% vs 17%, 95% CI 12–23%) of non-Hispanic children, *P* < 0.001), and children whose race and/or ethnicity differed from that of their parent (79%, 95% CI 60–91% vs 18%, 95% CI 13–25%) of children with the same race and ethnicity as their parent, *P* < 0.001). There was no association between child age and misidentification (odds ratio 1.04, 95% CI 0.99–1.11). No parents selected “I don’t know” or “I don’t want to say” on the questionnaire, and nine children had missing race and/or ethnicity data in the EHR. The results were not changed when these subjects were excluded in sensitivity analyses.

## DISCUSSION

Misidentification of child race and ethnicity was common in our PED, findings that remain similar to pediatric administrative-database analyses from the early 2000s.^8^ Our findings demonstrate a clear need to develop strategies to enhance precise data collection within our EHR and to facilitate self-report of race and ethnicity. Importantly, such efforts to improve precision must be partnered with analyses that consider complex demographic subgroups.^6^

Strategies applied in other healthcare systems have included the following: 1) staff training on self-report of race and ethnicity and education to increase patient awareness; 2) EHR systems that allow the selection of multiple races; 3) EHR alerts when race and/or ethnicity are missing; 4) use of granular race and ethnicity subcategories; and 5) a single-item question for race and ethnicity.^1^,^4^,^9^,^10^ Our findings highlight the importance of this multifaceted approach. All multiracial children in our sample were misidentified in the EHR. Our EHR allows for the selection of multiple races, yet in most of these cases multiracial children were misidentified in the EHR as having a single race. Additionally, by intentionally using a single item for race and ethnicity, we found that half of Hispanic parents did not select an additional race option for their child. Most of these children were categorized in the EHR as “other race,” an all-encompassing category that loses precision and is often excluded entirely from data analysis.^6^

We assessed race and ethnicity as a single construct for two reasons. First, as we found in our sample, individuals who identify as Hispanic may not additionally identify with a distinct race category.^12^,^13^ Second, this approach was pragmatic.^13^ Race and ethnicity are often presented as a single construct in health equity research, which requires researchers to collapse the two variable data that are found in administrative and hospital databases.^14^ Thus, our approach mirrors the practice of many health equity researchers. By offering choices that reflect the way data will be reported, we allow patients and parents greater self-determination in how precisely they will be identified.

## LIMITATIONS

This study is subject to limitations. Questionnaires were completed by parents, which may not reflect child self-identification. Race and ethnicity categories used by the US Census Bureau are themselves limited and do not fully capture individual realities. We were unable to determine how EHR data was collected and could not determine which misidentifications occurred at the level of data entry (ie, if race and ethnicity were determined by staff observation). Demographics may have been collected during prior visits within the hospital system, so our findings are not sufficient to identify misidentifications that are uniquely due to the PED registration process. Finally, we approached a convenience sample, and responses may have been influenced by the timing and methods of questionnaire administration. As part of a quality improvement initiative, our findings are not intended to be generalizable.

## CONCLUSION

Despite representing arbitrary social constructs, accurate race and ethnicity data are essential to identifying and addressing health inequities. Although we found that rigidity within race and ethnicity items in the EHR was an important factor in many misidentifications (ie, the requirement for both a race and ethnicity response), we also found that some features of the EHR were not used (ie, the ability to select multiple responses within a category). This work provides the basis for a multifaceted quality improvement effort at our institution. The quality of child race and ethnicity data in the emergency setting warrants further consideration across health equity efforts.

## Figures and Tables

**Table 1:** Patient characteristics stratified by accuracy of demographic data in electronic health records.

	Child race and ethnicity correct in EHR (n = 150)	Child race and/or ethnicity misidentified in EHR (n = 56)
Child age in years, median (IQR)	6 (1 – 12)	3 (1 – 8)
Child race and ethnicity as reported by parent, no. (%)		
Not Hispanic^1^		
American Indian/Alaska Native	0	0
Asian	0	1 (100)
Black/African American	50 (92.6)	4 (7.4)
Native Hawaiian/other Pacific Islander	0	1 (100)
White	96 (92.3)	8 (7.7)
Other	0	1 (100)
Multiracial	0	15 (100)
Hispanic^2^		
No race selected^3^	0	16 (100)
Black/African American	1 (20.0)	4 (80.0)
White	3 (37.5)	5 (62.5)
Multiracial	0	2 (100)
Child and parent race and/or ethnicity differ,^3^ no. (%)	6 (20.7)	23 (79.3)

1 Parent did not select “Hispanic” in the single combined race and ethnicity question.

2 Parent selected “Hispanic” in the single combined race and ethnicity question.

3 Child and parent race and ethnicity as reported by the parent.

*EHR*, electronic health record; IQR, interquartile range.

**Table 2:** Details of ethnicity listed in the electronic health record (EHR) compared to parent report of child race and ethnicity for children with misidentifications in the EHR.

Child race and ethnicity identified by the parent	EHR misidentification
Not Hispanic^2^	
Asian	“Other Race” (1)
Black/African American	“White” (2), “Other race” (1), “Unknown” (1)
Native Hawaiian/other Pacific Islander	Unknown (1)
White	“White + American Indian/Alaska Native” (AIAN) (1), “Asian” (1), “White + Asian + other” (1), “Other race” (1), “Declines to state” (4)
Other^3^	“White” (1)
Multiracial	
AIAN + White	“White” (2)
AIAN + Black + White	“Black/African American” (1), “Black/African American + White” (1)
Black/African American + White	“Black/African American” (3), “White” (3), “Other race” (1), “Unknown” (1), “Declines to state” (1)
Other^4^ + White	“White” (2)
Hispanic	
No race selected^5^	“White” (4), “White + other race” (1), “Other race” (11)
Black/African American	“Not Hispanic or Latino + Black/African American” (1), “Hispanic + other race” (2)
White	“Not Hispanic or Latino + White” (3), “Not Hispanic or Latino + other race” (1), “Hispanic + other race” (1)
Multiracial	
Black/African American + White	“Not Hispanic or Latino + Black/African American + White” (1), “Hispanic + other race” (1)

1 Number in parentheses indicates the number of children for each listed EHR race and ethnicity combination.

2 Ethnicity not specified, as all were correctly identified as “not Hispanic or Latino” in the EHR.

3 One parent wrote in “Black white mixed.”

4 One parent wrote in “Native American”; one parent wrote in “Indian.”

5 Ethnicity not specified as all were correctly identified as “Hispanic” in the EHR.

*EHR*, electronic health record; IQR, interquartile range.
